# Determinants of Unmet Healthcare Needs During the Final Stage of the COVID-19 Pandemic: Insights From a 21-Country Online Survey

**DOI:** 10.3389/ijph.2024.1607639

**Published:** 2024-10-28

**Authors:** Samuel Lewis, Louisa Ewald, Herbert C. Duber, Ali H. Mokdad, Emmanuela Gakidou

**Affiliations:** ^1^ Department of Emergency Medicine, University of Washington, Seattle, WA, United States; ^2^ Institute for Health Metrics and Evaluation, University of Washington, Seattle, WA, United States; ^3^ Department of Health Metrics Sciences, University of Washington, Seattle, WA, United States

**Keywords:** health system, preventive care, chronic care, COVID-19 pandemic, healthcare utilization

## Abstract

**Objectives:**

During the COVID-19 pandemic, essential health services experienced significant disruptions, impacting preventive and chronic care across the world.

**Methods:**

Utilizing the Pandemic Recovery Survey (PRS), conducted online with Facebook’s Active User Base across 21 countries between March and May 2023, this cross-sectional study identifies the magnitude of and key factors associated with unmet preventive and chronic care needs during the late stage of the COVID-19 pandemic.

**Results:**

Approximately 28.2% of respondents reported unmet preventive care needs, and 42.1% experienced unmet chronic care needs, with key determinants including food insecurity (aOR 1.94, 95% CI 1.81–2.07 for preventive services; aOR 1.85, 95% CI 1.68–2.03 for existing conditions) and distrust in health professionals (aOR 1.09, 95% CI 1.03–1.15 for preventive services; aOR 1.53, 95% CI 1.41–1.66 for existing conditions).

**Conclusion:**

The findings underscore a widespread unmet need for health services, highlighting the impact of social determinants and trust in health professionals on service disruption. The results suggest that pandemic recovery efforts should focus on the most affected groups to bridge health disparities and ensure an equitable recovery.

## Introduction

The impact of COVID-19 on healthcare systems and their capacity to deliver essential services has been profound. Research across a range of global contexts has reported widespread disruptions in health service use during the initial and peak phases of the pandemic [1, 2]. The scope and implications of care disruptions are vast, including missed childhood vaccinations, declines in maternal and newborn health service use, delayed cancer diagnoses, and foregone cancer care [2–6]. Initial estimates suggest a substantial direct resultant burden of morbidity and mortality [7, 8]. However, there remains a gap in understanding health service utilization during the late stage of the pandemic. As countries and health systems transition from acute crisis management to recovery, efforts are underway to restore essential health service provision. There is a need to better understand the determinants of health service disruption as well as the extent of its persistence in the current phase of the pandemic in order to inform a more effective recovery effort.

The current study aims to address this gap by analyzing health service utilization and its determinants during the late stages of the COVID-19 pandemic. This period marks a critical juncture, just before the World Health Organization’s declaration of the pandemic’s end. This study does not aim to assess the pandemic’s direct impact on health service utilization over its duration but focuses on the factors that shaped utilization during this late stage.

Research to date has demonstrated several individual-level factors associated with disruptions in healthcare since the onset of the pandemic. Studies from Europe and the US have reported higher levels of delayed or foregone care among women, individuals with financial insecurity and lower economic status, and poorer baseline health status [9–13]. In contrast, there have been mixed findings on the relationship between disruptions in care and age and education [9, 10, 14]. There is more limited evidence on individual determinants of care disruption in low- and middle-income countries, including studies from Thailand and South Africa with mixed findings on the association of socioeconomic status and disruptions in care [15, 16].

In investigating the determinants of health service disruption, two important topics warrant particular attention. First, exploring the role of social need is critical to understanding the impact of the pandemic on health inequities. Healthcare delays have previously been shown to be more prevalent among individuals of lower socioeconomic status [17]. The extent to which social factors including financial and food insecurity, education, and gender shaped health service utilization is a key factor in the pandemic’s overall impact on health disparities. Second, there is a need to further explore the role of trust in public health and healthcare providers. Trust has emerged as an important theme of the pandemic, mediating adherence to public health measures, care seeking, and vaccine uptake [18–21]. Unfortunately, the COVID-19 pandemic has coincided with an era of low global confidence in healthcare providers and systems, and the extent to which distrust has mediated care disruptions during the pandemic is incompletely understood.

This study uses data from the Pandemic Recovery Survey (PRS), an online survey administered in 21 countries between March and May 2023 to investigate health, economic, behavioral, and educational indicators affected by the COVID-19 pandemic. Online surveys have proven to be a valuable tool in public health research, enabling quick, cost-effective data collection across diverse geographical locations and populations [20, 22, 23]. These online platforms offer the agility needed to respond to health emergencies, collecting timely and relevant information. The wide reach and low cost of online surveys capture a broad spectrum of experiences, allowing for cross-regional and temporal comparisons. These surveys are particularly useful in understanding the real-world challenges faced by individuals, such as healthcare barriers or food insecurity, filling in significant gaps left by other research methodologies.

In this paper, we describe the use of essential health services during the late stage of the COVID-19 pandemic and explore individual-level determinants of unmet healthcare needs, with a focus on reported use of preventive services and care for existing chronic medical conditions. By deepening understanding of the complexities of healthcare delivery during the pandemic, we strive to mitigate the impacts of health service disruptions and inform future public health strategies and policies.

## Methods

### Study Design and Participants

This cross-sectional, internet-based survey was conducted as part of the Pandemic Recovery Survey (PRS) across 21 countries from March to May 2023 [24]. The PRS sought to capture the population-level impact of the COVID-19 pandemic, focusing on economic, educational, and health outcomes.

The study design and questionnaire were reviewed and approved by the Institutional Review Board of the University of Washington.

The target population included active Facebook users aged 18 and over in the selected 21 countries. Participants were identified through the Facebook Active User Base (FAUB) which was divided into strata by location and gender to help ensure a balanced coverage of genders in the final sample. Random samples were drawn from each gender strata and participants received an invitation via notification on their Facebook account. Participants were not able to take the survey twice or send the link to others.

The questionnaire was translated and checked by native speakers in 15 languages and data were collected through an online survey platform, Qualtrics. Researchers did not have access to any identifying information, including the participant’s Facebook information. Meta did not have access to any data.

The questionnaire was pilot tested with a percentage of the total sample in all countries before launch.

The 21 countries were chosen based on population, geographic distribution, number of active Facebook users, and estimated healthcare disruption due to the COVID-19 pandemic.

### Measures

Respondents were asked about utilization of preventive health services and care for existing chronic medical conditions in the preceding 6 months. Preventive health services included bloodwork, dental care, vision care, blood pressure screening, diabetes screening, cholesterol screening, hearing care, colonoscopy, mammogram, and Pap smear. Respondents who reported an existing medical condition (heart attack, stroke, high blood pressure, cancer, diabetes, lung disease, dementia or Alzheimer’s disease, mental health condition, addiction or substance use disorder, liver disease, and kidney disease) and currently needed care were asked if they had received care from a healthcare provider for their condition(s) in the previous 6 months. Respondents were able to answer that they had received care when needed, only received care some of the times needed, did not receive care when needed, or did not require care or treatment. For this study’s purposes, an unmet healthcare need is defined as respondents reporting a current healthcare need for a preventive service or chronic medical condition and that they did not receive care every time they needed it in the last 6 months.

Respondents were asked whether they felt healthcare professionals (such as physicians and nurses) were “very trustworthy, somewhat trustworthy, neither trustworthy nor untrustworthy, not very trustworthy, or not trustworthy at all.” Those reporting that they considered healthcare professionals very or somewhat trustworthy were categorized into the “trusts healthcare professionals” category, while the other three were categorized as not trusting healthcare professionals.

Demographic questions included age (18–24 years, 30–49 years, and 50 years and older), gender (male, female), educational attainment (primary or less, secondary, college or more), financial stability (easy to afford household expenses, somewhat difficult, or very difficult), and food security (enough to eat, sometimes enough to eat, or not enough to eat over preceding 30 days).

### Statistical Analysis

We performed descriptive analyses to describe demographic factors, COVID-19 vaccination status, food and financial security, and trust in health professionals by country. Demographics were analyzed with unweighted samples, with subsequent analyses performed using weighted sampling. We then describe care-seeking behavior and receipt of services in the prior 6 months for existing medical conditions and preventive care. Univariate and multivariate regression models were subsequently estimated to examine the predictors of unmet healthcare needs. We first assessed unmet preventive service needs, defined as receiving only some or none of the preventive services sought by respondents in the prior 6 months. Univariate and multivariate logistics regression models were estimated with predictors of age, gender, education, food security, financial security, COVID vaccination status, health status, and trust in health professionals. Unmet healthcare needs for an existing medical condition, defined as receiving only some or none of the needed care for an existing medical condition in the prior 6 months, was subsequently estimated with the above predictors, excluding health status. To further explore the role of trust in healthcare professionals, models estimating unmet healthcare need for existing medical conditions were performed at the country level to assess country-level variability. We conducted multivariable logistic regression with individuals nested within countries. Random effects were summarized with variance components, and country-specific random effects were reported relative to the global intercept. We analyzed the data using R software and the survey package [25].

## Results

### Study Description

Our analysis includes 426,584 individuals across 21 countries, with country-level sample sizes ranging from 9354 (Germany) to 40,613 (India). Among all respondents, 47.5% were female and 46.0% had a college education or higher, 44.0% had a secondary school education, and 10.0% had an education level of primary school or less. 34.3% of respondents were aged 18–29, 45.2% were aged 30–49, and 20.5% were aged 50 or older. Additional survey characteristics can be found in Supplementary Appendix Table 1A.

We compared unweighted survey characteristics and population estimates. Populations were categorized into location-age-gender buckets. Differences between the survey respondent proportions and the estimated population proportions were non-significant, with an average absolute difference of 2.8% and a maximum difference of 11.9%.

The proportion of individuals reporting one or more existing medical conditions ranged from 38.4% in Indonesia to 69.6% in the UK (51% overall) (Table 1). Self-reported COVID-19 vaccination rates (one or more reported vaccinations) ranged from 61.8% in South Africa to 96.8% in Viet Nam, with 86% of all respondents reporting one or more vaccinations. A majority of respondents reported receiving one or more lifetime preventive services in all survey countries, with rates ranging from 49.6% (India) to 82.1% (Germany) (61% overall). The most commonly received preventive services were Pap smear (69% among eligible women) and bloodwork (65% of respondents) (Supplementary Appendix).

**TABLE 1:** Proportion of respondents with known medical condition, COVID-19 vaccination, and lifetime utilization of preventive care services (data are from the Pandemic Recovery Survey, 21 countries, 2023).

Country	Any medical condition	At least one COVID-19 vaccination	Lifetime preventive service utilization
Argentina	51.5% (SE: 0.7%)	92% (SE: 0.5%)	78.4% (SE: 0.7%)
Brazil	61.3% (SE: 0.6%)	95.1% (SE: 0.3%)	75.3% (SE: 0.6%)
Chile	61.3% (SE: 0.9%)	94.4% (SE: 0.5%)	76.8% (SE: 0.9%)
Colombia	45.2% (SE: 1.2%)	86.8% (SE: 0.9%)	75.8% (SE: 1.1%)
Egypt	54.6% (SE: 1.1%)	68% (SE: 1.3%)	54.7% (SE: 1%)
Germany	69.2% (SE: 0.9%)	89.4% (SE: 0.6%)	82.1% (SE: 0.8%)
India	46.1% (SE: 0.4%)	93.3% (SE: 0.3%)	49.6% (SE: 0.3%)
Indonesia	38.4% (SE: 0.7%)	86.3% (SE: 0.6%)	66.8% (SE: 0.6%)
Italy	61.9% (SE: 0.9%)	94.3% (SE: 0.5%)	79.6% (SE: 0.8%)
Japan	61.7% (SE: 0.8%)	88.3% (SE: 0.6%)	85.7% (SE: 0.7%)
Mexico	48.6% (SE: 1.2%)	87.2% (SE: 1.1%)	72.9% (SE: 1%)
Nigeria	42.2% (SE: 0.8%)	48.5% (SE: 0.9%)	57% (SE: 0.8%)
Peru	52.2% (SE: 1.6%)	92.8% (SE: 1.1%)	69.5% (SE: 1.5%)
Philippines	44.5% (SE: 0.9%)	87.6% (SE: 1%)	58.8% (SE: 0.9%)
Poland	68.2% (SE: 1%)	75.4% (SE: 1.1%)	77.5% (SE: 1%)
South Africa	50.5% (SE: 1.3%)	61.8% (SE: 1.7%)	62.3% (SE: 1.2%)
Spain	61.8% (SE: 1.2%)	92.6% (SE: 1%)	76.8% (SE: 1%)
Türkiye	53.6% (SE: 1.3%)	85% (SE: 1.2%)	66.3% (SE: 1.2%)
UK	69.6% (SE: 1.4%)	89.1% (SE: 1.2%)	77% (SE: 1.1%)
United States	65.6% (SE: 0.8%)	76.3% (SE: 0.8%)	78.9% (SE: 0.7%)
Viet Nam	54.5% (SE: 1.4%)	96.8% (SE: 1%)	52.1% (SE: 1.2%)

### Health Service Seeking and Use in Preceding Six Months

**FIGURE 1:**
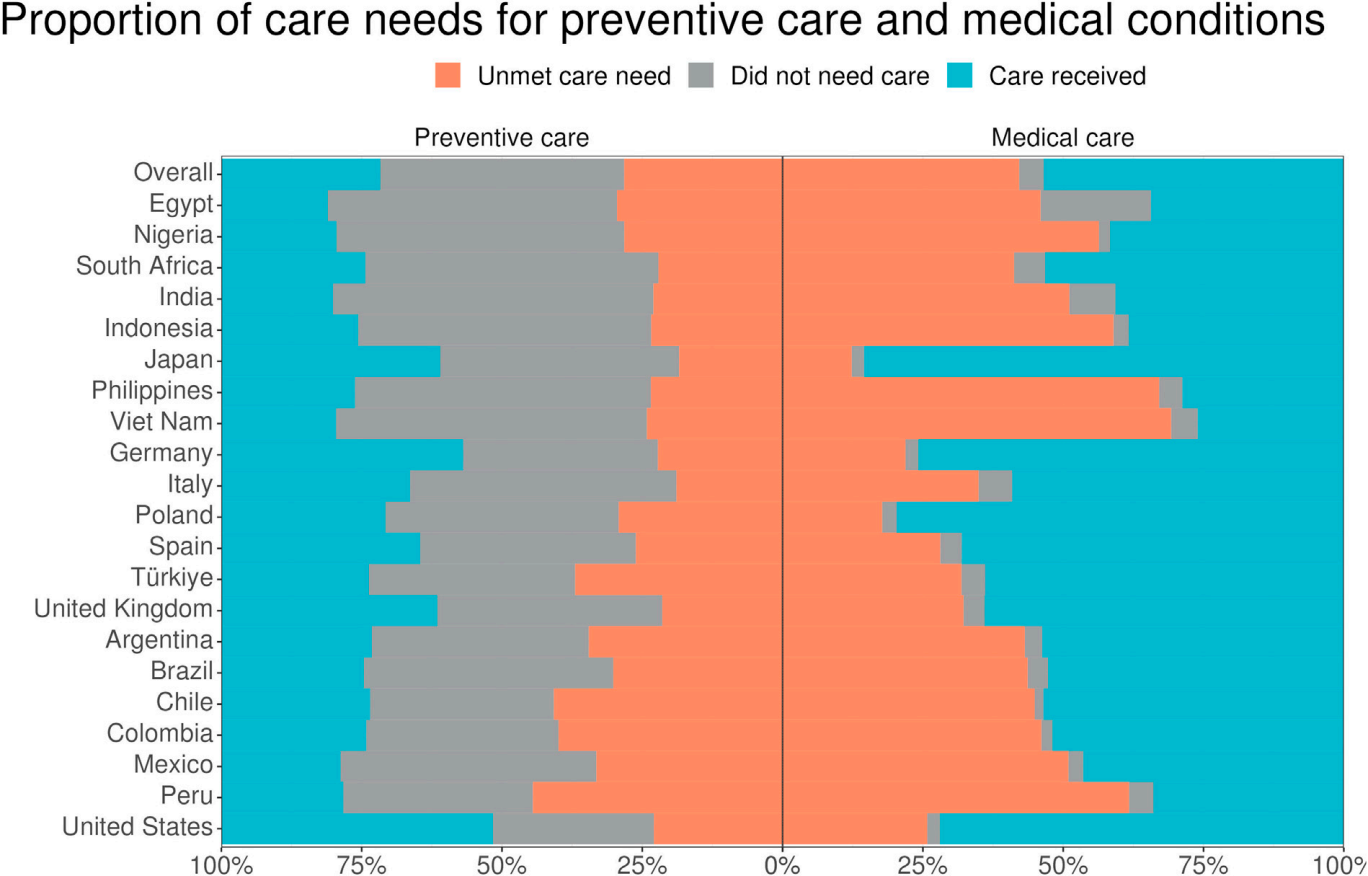
Proportion of care seeking for preventive service and existing medical conditions across 21 countries. A bar graph displaying the proportion of respondents seeking preventive and medical care by country and overall in the last 6 months (data are from the Pandemic Recovery Survey, 21 countries, 2023).

On average, 56.5% of all study respondents reported seeking one or more preventive health services in the preceding 6 months, ranging from 42.9% in India to 71.2% in the United States (Figure 1). Among all respondents, 28.2% reported an unmet preventive service need, with country-level rates ranging from 18.4% in Japan to 44.5% in Peru.

Among the 51% of respondents with an existing medical condition, 95.6% reported seeking care for their condition(s) in the preceding 6 months (Figure 1). Care-seeking rates at the country level ranged from 80.0% in Egypt to 98.2% in Nigeria. Overall, 42.1% reported an unmet care need for an existing condition, with country-level rates ranging from 12.3% in Japan to 69.2% in Viet Nam.

### Determinants of Unmet Preventive Service Needs

**FIGURE 2:**
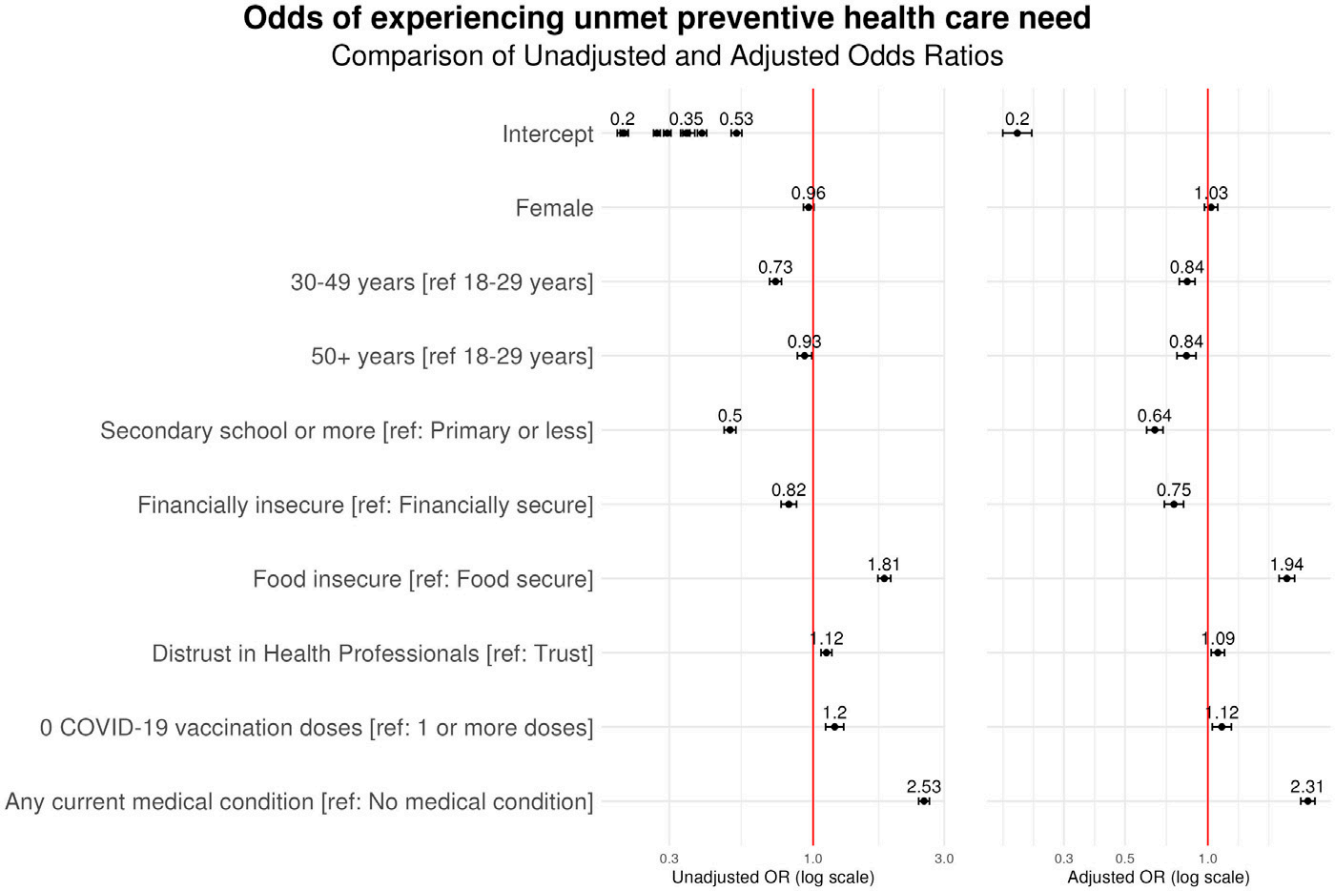
Determinants of unmet care need for preventive services across 21 countries. A forest plot displaying the odds ratios of a respondent experiencing an unmet preventive care need in preceding 6 months (data are from the Pandemic Recovery Survey, 21 countries, 2023).

Pooled unadjusted and adjusted regression analyses of unmet preventive service needs are shown in Figure 2. The individual-level characteristics most strongly associated with experiencing an unmet need for preventive services were food insecurity (aOR 1.94, 95% CI 1.81–2.07) and having one or more existing medical conditions (aOR 2.31, 95% CI 2.18–2.45). Unmet preventive service need was also positively associated with unvaccinated status (aOR 1.12, 95% CI 1.04–1.22) and distrust in health professionals (aOR 1.09, 95% CI 1.03–1.15). In contrast to food insecurity, financial insecurity was negatively associated with unmet preventive service need in the multivariate model (aOR 0.75, 95% CI 0.69–0.82). Those with a secondary or greater education level (aOR 0.64, 95% CI 0.60–0.69) and older age were also less likely to experience unmet preventive care needs (aOR 0.84 for both age groups 30–49 and 50+, relative to 18–29). There was no significant association observed between unmet preventive care needs and female gender (aOR 1.03, 95% CI 0.97–1.09). With the exception of gender in the adjusted model, all associations were statistically significant in both models.

### Determinants of Unmet Care Need for Existing Condition

**FIGURE 3:**
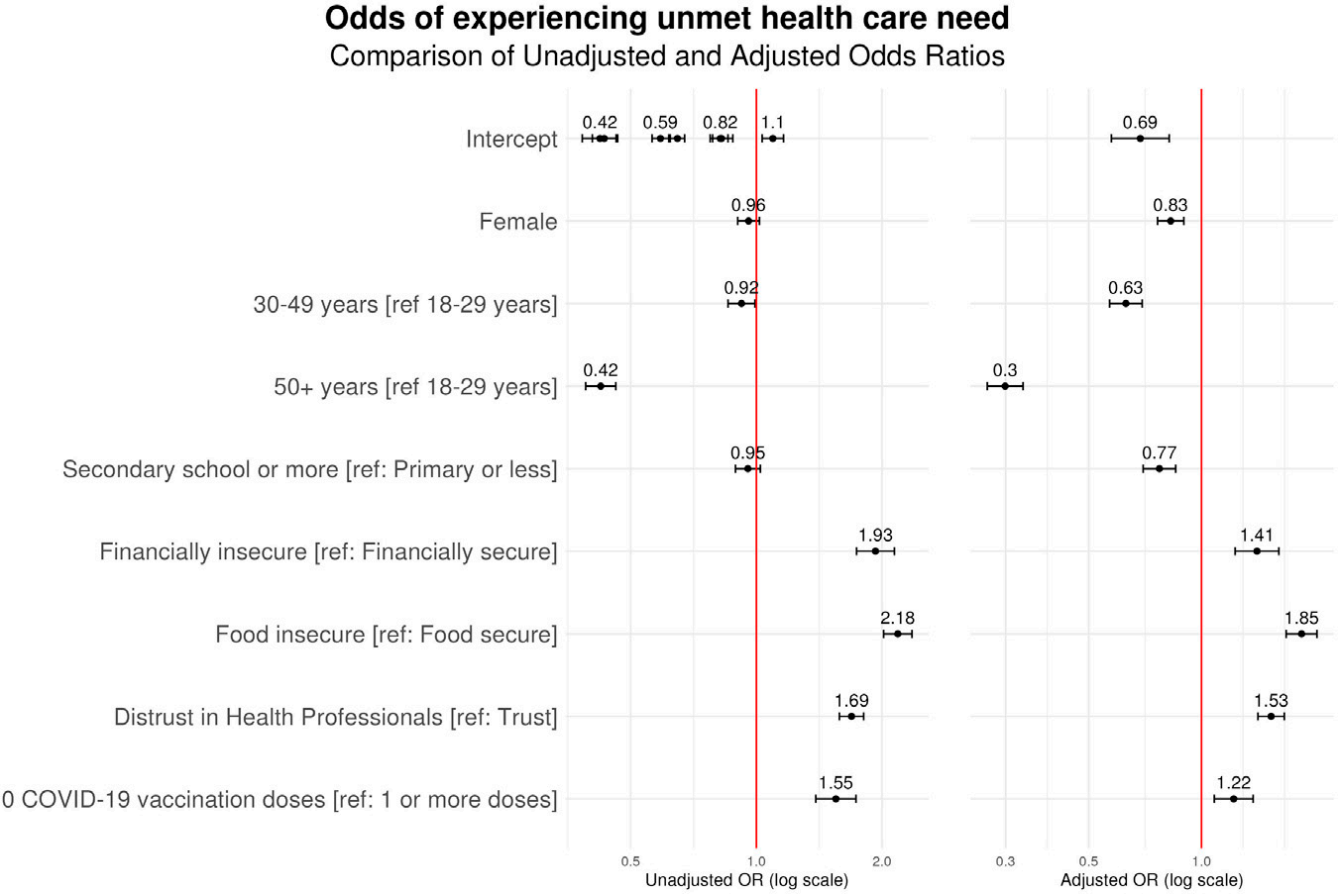
Determinants of unmet care need for chronic medical condition across 21 countries. A forest plot displaying the odds ratios of a respondent experiencing an unmet chronic medical condition need in the preceding 6 months (data are from the Pandemic Recovery Survey, 21 countries, 2023).

The determinants of unmet care need for an existing medical condition are depicted in Figure 3. Distrust in health professionals was significantly associated with unmet care needs, with an adjusted odds ratio of 1.53 relative to those with extremely or very trustworthy views of health professionals (95% CI 1.41–1.66). Individuals who reported food insecurity (aOR 1.85, 95% CI 1.68–2.03), financial insecurity (aOR 1.41, 95% CI 1.23–1.61), and being unvaccinated for COVID-19 (aOR 1.22, 95% CI 1.08–1.37) were also significantly more likely to experience an unmet care need for an existing condition. In contrast, characteristics associated with lower odds of experiencing an unmet care need include female gender (aOR 0.83, 95% CI 0.76–0.90), older age (age 30–49 years: aOR 0.63, 95% CI 0.57–0.69; age ≥50: aOR, 0.3, 95% CI 0.27–0.33), and higher education (aOR 0.877, 95% CI 0.7–0.85). All associations were statistically significant in both univariate and multivariate models, excluding female gender and higher education, which demonstrated significance only in the multivariate analysis.

### Country-Level Variation in Determinants of Unmet Care Needs

**FIGURE 4:**
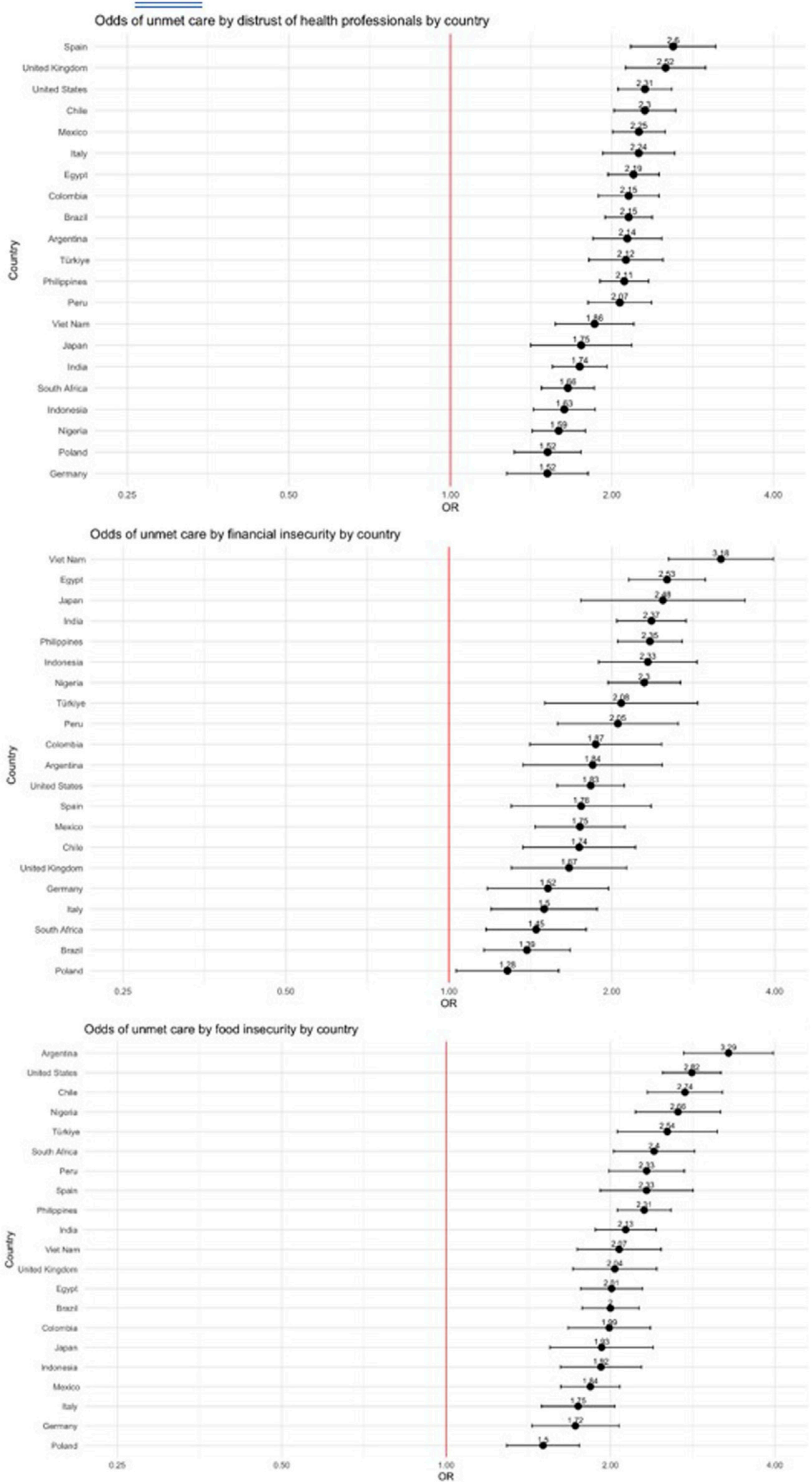
Country-level associations between **(A)** distrust in health professionals, **(B)** financial insecurity, and **(C)** food insecurity and unmet care need for chronic medical condition. Forest plots displaying the odds ratio of each country between three determinants (distrust in health professionals, financial insecurity, and food insecurity) and unmet chronic medical condition care (data are from the Pandemic Recovery Survey, 21 countries, 2023).

Multivariate regression analysis of unmet care need for an existing condition were performed at the country level to further explore the role of distrust in health professionals and socioeconomic factors. The adjusted odds ratios for distrust in health professionals, financial insecurity, and food insecurity are depicted in Figure 4. In all countries, distrust in health professionals was positively and significantly associated with experiencing an unmet care need, with the magnitude of association strongest in Spain (aOR 2.60, 95% CI 2.17–3.12). Similarly, both financial and food insecurity were positively and significantly associated with experiencing an unmet care need across all study countries, with the strongest magnitude of association in Viet Nam (aOR 3.18, 95% CI 2.54–3.97) and Argentina (aOR 3.29, 95% CI 2.72–3.97), respectively.

## Discussion

This study provides important insights into the impact of the COVID-19 pandemic on health service utilization. The study’s large sample size and inclusion of 21 diverse countries provides a uniquely broad perspective on the state of healthcare use globally in the late-stage pandemic. Though the online survey methodology carries limitations, including potential self-selection bias, unrepresentative samples, and incalculable response rates, it serves as a powerful tool for cross-country comparison and detection of larger international trends. The analysis identifies food insecurity, distrust in health professionals, and unvaccinated status as significant determinants of unmet healthcare need for both preventive services and chronic medical conditions. Conversely, higher education appears to serve as a protective factor against such unmet needs. Notably, the association between unmet care need and distrust in health professionals remains strong across all countries involved in the study, highlighting the widespread impact on health service disruption during the late stage of the COVID-19 pandemic.

One of the key findings from this analysis is the role of trust in healthcare professionals. Previous research has demonstrated that trust in health professionals and institutions served as a key mediator of care-seeking behavior during the pandemic [20]. The robust relationship, which is held in every study country, suggests that trust is essential to facilitating health system engagement and use during health system crises. Efforts to foster trust in governmental public health institutions and healthcare professionals could be key to maintaining continuity of care.

Furthermore, the study demonstrates a significant socioeconomic gradient in unmet care needs. Previous studies have found food insecurity to be one of the most important social determinants with known connections to poor health outcomes, largely through the development of chronic conditions and higher healthcare spending [26]. Interestingly, financial insecurity was negatively associated with unmet need for preventive services after controlling for covariates, despite evidence suggesting cost as the main barrier to care [27]. We hypothesize that national health insurance programs and other measures seeking to increase access to primary preventive services for low-income individuals may have served to mitigate economic access barriers. In the current study individuals with financial insecurity were more likely to report being unable to access care for their existing condition. Collectively, our findings indicate that previously vulnerable groups were most affected by disruptions in essential health services. We recommend that ongoing pandemic recovery efforts prioritize reducing barriers to essential health services among financially and socially disadvantaged populations.

The high overall rates of care seeking observed in this study, with over 95% of all respondents with chronic conditions seeking care, offer a positive outlook on healthcare seeking behavior during the late stage of the COVID-19 pandemic. Access to these services, however, was highly variable across countries, reflecting both uneven recoveries from the pandemic and pre-existing discrepancies in health service availability. Care seeking for preventive services was lower overall but still exceeded 50% of respondents in most countries. The findings from the current study indicate a positive return to higher rates of care seeking behavior as the global community recovers from the worst phases of the pandemic.

Lastly, our study suggests that COVID-19 vaccination status influences the likelihood of experiencing unmet healthcare needs for existing conditions. Studies have found that vaccinated individuals are more likely to seek care, and declines in delayed care are stronger among those with low socioeconomic backgrounds [28, 29]. It is plausible that vaccination may have increased individual willingness and ability to safely seek needed healthcare services for their medical conditions, although reverse causality (i.e., those receiving health services had better access to the COVID-19 vaccination) or unobserved confounding characteristics such as health literacy or motivation cannot be excluded from the present analysis.

As health systems continue to recover and draw lessons from the pandemic, they may look to those nations with relative preservation of access to essential health services for guidance. Our findings suggest that those with existing medical conditions may have leveraged pre-existing engagement with the healthcare system to reduce disruptions in care. The current study also demonstrates that delays in care during the pandemic fell along pre-existing socioeconomic lines, and that proactive efforts are needed to avoid worsening of health inequities.

### Limitations

Interpretation of the present study is limited by several factors. One, as an internet-based survey, there may be self-selection biases in survey participation, leading to unrepresentative samples and findings that do not reflect the larger population. As an online survey accessed through social media, it may reflect a more educated, literate, and young subset of the general population. Second, there were moderately high rates of incomplete surveys and resultant missing data. Despite the large overall sample size, biases introduced by non-random missing data cannot be excluded. Additionally, due to the survey methodology, response rates were unable to be calculated. Finally, the survey is a cross-sectional analysis that captures only recent health service utilization during the late phase of the COVID-19 pandemic. The current study did not assess trends in health service utilization over time, or the initial impact of the pandemic.

### Conclusion

This study has highlighted the integral role of trust in healthcare professionals and healthcare utilization. Additionally, the socioeconomic disparities in unmet healthcare needs, particularly food insecurity and educational attainment, demand targeted interventions to mitigate the widening gap in health inequities.

The study’s findings call for continued efforts to prioritize equitable access to essential and preventive health services and reinforce the importance of trust, equality, and recovery in ensuring effective global healthcare delivery. Additionally, the study also underscores the need for further research to address potential biases and expand on the comparative analysis of determinants of healthcare utilization across different populations.
