# Supplementary material for: Determinants of Unmet Healthcare Needs During the Final Stage of the COVID-19 Pandemic: Insights From a 21-Country Online Survey
Source: Int J Public Health. 2024 Oct 28;69:1607639. doi: 10.3389/ijph.2024.1607639 (PMC11550971; doi:10.3389/ijph.2024.1607639)
Supplement: Supplementary file 1 [file DataSheet1.docx]

**APPENDIX**

**Table A1. Unweighted survey sample characteristics**

|  | Argentina | Brazil | Chile | Colombia | Egypt | Germany | India | Indonesia | Italy | Japan |
| --- | --- | --- | --- | --- | --- | --- | --- | --- | --- | --- |
| n | 12,716 | 22,115 | 12,001 | 17,942 | 30,778 | 9354 | 40,613 | 29,571 | 17,487 | 9978 |
| Age (%) |  |  |  |  |  |  |  |  |  |  |
| 18–29 years | 3348 (26.3) | 5098 (23.1) | 2042 (17.0) | 7279 (40.6) | 14,351 (46.6) | 962 (10.3) | 17,004 (41.9) | 12,496 (42.3) | 1716 (9.8) | 300 (3.0) |
| 30–49 years | 5775 (45.4) | 11,123 (50.3) | 5660 (47.2) | 7848 (43.7) | 13,363 (43.4) | 4284 (45.8) | 20,126 (49.6) | 14,335 (48.5) | 7825 (44.7) | 2866 (28.7) |
| 50+ years | 3593 (28.3) | 5894 (26.7) | 4299 (35.8) | 2815 (15.7) | 3064 (10.0) | 4108 (43.9) | 3483 (8.6) | 2740 (9.3) | 7946 (45.4) | 6812 (68.3) |
| Education (%) |  |  |  |  |  |  |  |  |  |  |
| College or more | 3079 (24.2) | 5807 (26.3) | 4500 (37.5) | 5962 (33.2) | 20,626 (67.0) | 2472 (26.4) | 30,313 (74.6) | 8139 (27.5) | 5081 (29.1) | 5178 (51.9) |
| Primary school or less | 3120 (24.5) | 8165 (36.9) | 1604 (13.4) | 2362 (13.2) | 1238 (4.0) | 481 (5.1) | 3298 (8.1) | 3793 (12.8) | 2032 (11.6) | NR |
| Secondary school | 6517 (51.3) | 8143 (36.8) | 5897 (49.1) | 9618 (53.6) | 8914 (29.0) | 6401 (68.4) | 7002 (17.2) | 17,639 (59.6) | 10,374 (59.3) | 4729 (47.4) |
| Gender (%) | |  |  |  |  |  |  |  |  |  |
| Female | 5948 (46.8) | 10,584 (47.9) | 6045 (50.4) | 8705 (48.5) | 12,417 (40.3) | 5050 (54.0) | 18,575 (45.7) | 13,557 (45.8) | 9828 (56.2) | 4270 (42.8) |
| Male | 6570 (51.7) | 11,391 (51.5) | 5789 (48.2) | 9021 (50.3) | 17,943 (58.3) | 4195 (44.8) | 21,804 (53.7) | 15,654 (52.9) | 7422 (42.4) | 5580 (55.9) |
| Prefer not to answer or non-binary | 198 (1.6) | 140 (0.6) | 167 (1.4) | 216 (1.2) | 418 (1.4) | 109 (1.2) | 234 (0.6) | 360 (1.2) | 237 (1.4) | 128 (1.3) |

NR: Not reportable due to sample size limitations

**Table A1 cont. Unweighted survey sample characteristics**

|  | Mexico | Nigeria | Peru | Philippines | Poland | South Africa | Spain | Türkiye | UK | USA | Viet Nam |
| --- | --- | --- | --- | --- | --- | --- | --- | --- | --- | --- | --- |
| n | 22,965 | 27,358 | 16,360 | 38,977 | 16,249 | 24,982 | 9408 | 12,859 | 8213 | 10,629 | 25,400 |
| Age (%) |  |  |  |  |  |  |  |  |  |  |  |
| 18–29 years | 8376 (36.5) | 12,868 (47.0) | 6090 (37.2) | 16,707 (42.9) | 4854 (29.9) | 9603 (38.4) | 1044 (11.1) | 1597 (12.4) | 1093 (13.3) | 1699 (16.0) | 14,204 (55.9) |
| 30–49 years | 10,536 (45.9) | 12,545 (45.9) | 7001 (42.8) | 17,823 (45.7) | 6118 (37.7) | 12,100 (48.4) | 4199 (44.6) | 7049 (54.8) | 3291 (40.1) | 4851 (45.6) | 9177 (36.1) |
| 50+ years | 4053 (17.6) | 1945 (7.1) | 3269 (20.0) | 4447 (11.4) | 5277 (32.5) | 3279 (13.1) | 4165 (44.3) | 4213 (32.8) | 3829 (46.6) | 4079 (38.4) | 2019 (7.9) |
| Education (%) |  |  |  |  |  |  |  |  |  |  |  |
| College or more | 10,150 (44.2) | 18,231 (66.6) | 7211 (44.1) | 20,636 (52.9) | 5196 (32.0) | 8956 (35.8) | 3459 (36.8) | 4304 (33.5) | 4743 (57.7) | 5820 (54.8) | 11,288 (44.4) |
| Primary school or less | 1366 (5.9) | 706 (2.6) | 915 (5.6) | 2779 (7.1) | 1380 (8.5) | 1256 (5.0) | 2065 (21.9) | 2346 (18.2) | 342 (4.2) | 796 (7.5) | 1634 (6.4) |
| Secondary school | 11,449 (49.9) | 8421 (30.8) | 8234 (50.3) | 15,562 (39.9) | 9673 (59.5) | 14,770 (59.1) | 3884 (41.3) | 6209 (48.3) | 3128 (38.1) | 4013 (37.8) | 12,478 (49.1) |
| Gender (%) | |  |  |  |  |  |  |  |  |  |  |
| Female | 11,207 (48.8) | 9527 (34.8) | 7898 (48.3) | 19,919 (51.1) | 8923 (54.9) | 13,105 (52.5) | 5197 (55.2) | 5279 (41.1) | 4197 (51.1) | 6011 (56.6) | 11,492 (45.2) |
| Male | 11,362 (49.5) | 17,708 (64.7) | 8309 (50.8) | 17,970 (46.1) | 7125 (43.8) | 11,585 (46.4) | 4110 (43.7) | 7491 (58.3) | 3913 (47.6) | 4386 (41.3) | 13,344 (52.5) |
| Prefer not to answer or non-binary | 396 (1.7) | 123 (0.4) | 153 (0.9) | 1088 (2.8) | 201 (1.2) | 292 (1.2) | 101 (1.1) | NR | 103 (1.3) | 232 (2.2) | 564 (2.2) |

NR: Not reportable due to sample size limitations

**Table A2. Adjusted and unadjusted coefficients in the preventive care model**

| **Term** | **Unadjusted odds ratio (95% CI) *** | **Adjusted odds ratio (95% CI) *** |
| --- | --- | --- |
| Female | 0.96 (0.92, 1.01)* | 1.03 (0.97, 1.09) |
| 30–49 years | 0.73 (0.69, 0.77) *** | 0.84 (0.79, 0.9) *** |
| 50+ years | 0.93 (0.88, 0.99) ** | 0.84 (0.77, 0.91) *** |
| Secondary or more | 0.50 (0.47, 0.52) *** | 0.64 (0.60, 0.69) *** |
| Financially insecure | 0.82 (0.76, 0.87) *** | 0.75 (0.69, 0.82) *** |
| Food insecure | 1.81 (1.72, 1.91) *** | 1.94 (1.81, 2.07) *** |
| Distrusts health professionals | 1.12 (1.07, 1.17) *** | 1.09 (1.03, 1.15) ** |
| Zero COVID-19 vaccination doses | 1.2 (1.11, 1.29) *** | 1.12 (1.04, 1.22) ** |
| Any medical condition | 2.53 (2.42, 2.65) *** | 2.31 (2.18, 2.45) *** |

*P-values are displayed as *** >0.001, ** >0.05, *>0.1

**Table A3. Adjusted and unadjusted coefficients for unmet medical care model**

| **Term** | **Unadjusted odds ratio (95% CI) *** | **Adjusted odds ratio (95% CI) *** |
| --- | --- | --- |
| Female | 0.96 (0.9, 1.02) | 0.83 (0.76, 0.9) *** |
| 30–49 years | 0.92 (0.86, 0.99) ** | 0.63 (0.57, 0.69) *** |
| 50+ years | 0.42 (0.39, 0.46) *** | 0.3 (0.27, 0.33) *** |
| Secondary or more | 0.95 (0.89, 1.02) | 0.77 (0.7, 0.85) *** |
| Financially insecure | 1.93 (1.74, 2.14) *** | 1.41 (1.23, 1.61) *** |
| Food insecure | 2.18 (2.02, 2.36) *** | 1.85 (1.68, 2.03) *** |
| Distrusts health professionals | 1.69 (1.58, 1.81) *** | 1.53 (1.41, 1.66) *** |
| Zero COVID-19 vaccination doses | 1.55 (1.39, 1.73) *** | 1.22 (1.08, 1.37) ** |

*P-values are displayed as *** >0.001, ** >0.05, *>0.1
